# Beneficial Effects of Remifentanil Against Excitotoxic Brain Damage in Newborn Mice

**DOI:** 10.3389/fneur.2019.00407

**Published:** 2019-04-24

**Authors:** Clément Chollat, Maryline Lecointre, Matthieu Leuillier, Isabelle Remy-Jouet, Jean-Claude Do Rego, Lénaïg Abily-Donval, Yasmina Ramdani, Vincent Richard, Patricia Compagnon, Bertrand Dureuil, Stéphane Marret, Bruno José Gonzalez, Sylvie Jégou, Fabien Tourrel

**Affiliations:** ^1^INSERM U1245, Genetics and Pathophysiology of Neurodevelopment Disorders Team, Faculty of Medicine, Institute of Research and Innovation in Biomedicine, Normandy University, Rouen, France; ^2^Neonatal Intensive Care Unit of Port-Royal, Paris Centre University Hospitals, APHP, Paris Descartes University, Paris, France; ^3^INSERM U1096, Biology Oxidative Stress Systems Platform, Institute for Research and Innovation in Biomedicine, Normandy University, Rouen, France; ^4^Platform of Behavioural Analysis (SCAC), Faculty of Medicine, Rouen, France; ^5^Department of Neonatal Pediatrics and Intensive Care, Rouen University Hospital, Rouen, France; ^6^Department of Pharmacology, Rouen University Hospital, Rouen, France; ^7^Department Anesthetics and Intensive Care, Rouen University Hospital, Rouen, France

**Keywords:** anesthetics, preterm birth, ibotenate, neurotoxicity, cell death, inflammation, behavioral test

## Abstract

**Background:** Remifentanil, a synthetic opioid used for analgesia during cesarean sections, has been shown in *ex vivo* experiments to exert anti-apoptotic activity on immature mice brains. The present study aimed to characterize the impact of remifentanil on brain lesions using an *in vivo* model of excitotoxic neonatal brain injury.

**Methods:** Postnatal day 2 (P2) mice received three intraperitoneal injections of remifentanil (500 ng/g over a 10-min period) or saline just before an intracortical injection of ibotenate (10 μg). Cerebral reactive oxygen species (ROS) production, cell death, *in situ* labeling of cortical caspase activity, astrogliosis, inflammation mediators, and lesion size were determined at various time points after ibotenate injection. Finally, behavioral tests were performed until P18.

**Results:** In the injured neonatal brain, remifentanil significantly decreased ROS production, cortical caspase activity, DNA fragmentation, interleukin-1β levels, and reactive astrogliosis. At P7, the sizes of the ibotenate-induced lesions were significantly reduced by remifentanil treatment. Performance on negative geotaxis (P6-8) and grasping reflex (P10-12) tests was improved in the remifentanil group. At P18, a sex specificity was noticed; remifentanil-treated females spent more time in the open field center than did the controls, suggesting less anxiety in young female mice.

**Conclusions:** *In vivo* exposure to remifentanil exerts a beneficial effect against excitotoxicity on the developing mouse brain, which is associated with a reduction in the size of ibotenate-induced brain lesion as well as prevention of some behavioral deficits in young mice. The long-term effect of neonatal exposure to remifentanil should be investigated.

## Introduction

The potential neurotoxicity of anesthetics in the perinatal period prompted the US Food and Drug Administration (FDA) to release a drug safety communication warning in 2016 that stated, “repeated or lengthy use of general anesthetics or sedation drugs during surgeries or procedures in children younger than 3 years of age or in pregnant women during the final trimester may affect development of children's brains” (1). Use of anesthetics in the perinatal period is, therefore, a particularly delicate matter, and obstetric anesthesiologists have to weigh risks and benefits in order to make decisions regarding the exposure of the developing brain to anesthetics; on the one hand, it is ethically essential to relieve pain of the parturient during labor or cesarean section, while on the other hand, anesthetics, including opioids, are potentially deleterious to brain development when used during the fetal period or before 3 years of age (2). Indeed, exposure to anesthetics is associated with altered neuronal proliferation and degeneration as well as behavioral disturbances in animal studies (3), and leads to early alterations in cerebral structure and short-term neurobehavioral problems in human studies (4, 5). Otherwise, human preterm neonates exhibit cerebral vulnerability that can lead to encephalopathy during preterm birth, which can cause neurological morbidities such as cognitive and behavioral disabilities, sensorimotor deficiencies, and cerebral palsy (6). Therefore, it seems crucial to study the impact of anesthetics on the immature brain, especially through animal pre-clinical studies.

Among the anesthetics used in the perinatal period in humans, remifentanil is an opioid with attractive pharmacokinetic properties. It is a synthetic ultrashort-acting opioid, a potent mu-opioid receptor agonist, is metabolized by non-specific plasma and tissue esterases, and is consequently unaffected by renal or liver function (7). Due to these pharmacologic properties, the use of remifentanil for the induction of general anesthesia in cesarean delivery or during labor has recently been proposed to reduce potential side effects of maternal anesthesia on the newborn, such as neonatal apnea or the need for mechanical ventilation at birth (8–10). However, this use of remifentanil raises the question of its impact on the developing brain, as it readily crosses the placenta (11). In humans, remifentanil is known to induce postoperative secondary hyperalgesia involving the N-methyl-D-aspartate receptor (NMDA-R) (12). Pre-clinical studies evaluating the hyperalgesia model showed that activation of the mu-opioid receptor by remifentanil induces a phosphorylation cascade involving phosphorylation of the GluN2B subunit of the NMDA-R via protein kinase C (13–17). Through this hyperalgesia model, it has been shown that remifentanil indirectly interacts with the NMDA-R. However, in the developing brain, activation of the NMDA-R can lead to excitotoxic and anti-apoptotic effects, depending on the cortical layer (18). Using an *ex vivo* model of brain slices from postnatal day 2 mice (P2), we previously showed that remifentanil exerts anti-apoptotic activity without a necrotic effect by interacting with the NMDA-R and the intrinsic mitochondrial-dependent apoptotic pathway, which are two major actors of the excitotoxic cascade (19). The aim of our present study was to evaluate the impact of remifentanil on the developing brain *in vivo* to assess its potential neurotoxicity using a well-defined rodent model of neonatal brain lesions by intracortical injection of the NMDA-R agonist ibotenate (20, 21). Intracortical administration of ibotenate in P2 mice can reproduce some aspects of perinatal brain lesions observed in human preterm neonates, such as periventricular leukomalacia, around 26 weeks of gestation (22). In our experiments, remifentanil administration preceded the lesion to reproduce the chronology observed in clinical practice, namely, fetal exposure to remifentanil during general anesthesia for cesarean sections before the onset of excitotoxic brain lesions related to preterm birth. We investigated the effects of remifentanil in P2 mice in the context of excitotoxicity on (1) brain reactive oxygen species (ROS) production, cell death, astrogliosis, inflammation mediators, and the size of ibotenate-induced lesions, and (2) sensorimotor development and motor performance starting in the neonatal period.

## Methods

Experimental procedures were consistent with the Animal Research: Reporting *in vivo* Experiments (ARRIVE) guidelines. Researchers were completely blinded to the experimental groups through numerical sample-marking with the researchers being unaware of the group in order to avoid bias.

### Experimental Design

**Figure 1:**
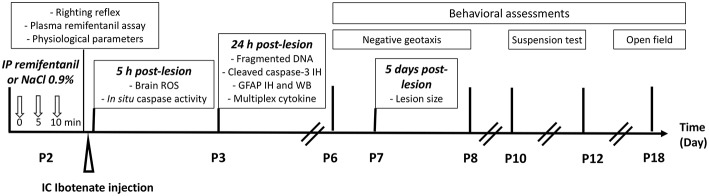
Experimental design.

First, we determined an effective dose of remifentanil for inducing sedation in P2 pups using the righting reflex test, determining the plasma remifentanil level, and quantifying physiological parameters such heart and ventilation rates and blood gas. Ibotenate administration was performed at P2 on the basis that ibotenate induced periventricular white matter cystic lesions (including laminar neuronal losses, abnormal sulcation, and neuronal ectopias), closely mimicking several aspects of human cystic periventricular leukomalacia observed in human preterm neonates at 26 weeks of gestation (23). Following ibotenate injection, the pup brain undergoes a series of cellular and molecular changes ranging from inflammatory changes such as gliosis and cytokine production within a few hours of the insult to neuronal loss within a few days, and the subsequent formation of a lesion (23, 24). The different time points used in the present study for each assay were justified by different activation kinetics in the excitotoxic cascade (25), defined from several previous *ex vivo* and *in vivo* studies (18). *Ex vivo* studies supported that the optimal time point to quantify caspase-3 cleavage and caspase activity was 6 h after lesion formation, and that caspase-3 activity was markedly decreased within the first 24 h (18). ROS are rapidly produced after an excitotoxic lesion, and just precedes caspase activation. Thus, ROS were measured 10 min and 5 h after ibotenate injection (25). DNA fragmentation, which leads to cell death through apoptosis or necrosis, is usually measured 24 h after a lesion forms, whereas it is not possible to quantify it 5 days postlesion (22, 26, 27). Levels of glial fibrillary acidic protein (GFAP) and inflammatory cytokines were evaluated 24 h after ibotenate injection, since gliosis and inflammation processes occur early after a cerebral lesion and peak within 12-24 h, before rapidly decreasing 48–72 h after lesion formation (22, 28). Ibotenate-induced lesions were measured 5 days after ibotenate administration, as described by Marret et al. (23). Negative geotaxis and grip tests were performed from P6 to P8 and P10 to P12, respectively, and locomotor activity and exploration tests were performed at P18. The timing of these experiments was based on previous behavioral studies and depends on the neuromotor development of the mice (29–31). The study design is described in Figure 1.

### Animals

Naval Medical Research Institute (NMRI) mice (Janvier, Le Genest Saint Isle, France) were housed under controlled conditions (12-h light/dark cycle, at 21 ± 1°C with free access to food and tap water). Animal manipulations were performed in accordance with the European Communities Council Directives (86/609/EEC) and the French National legislation (ethical approval nos. 01316.02 and 01680.02). Females were individually housed and inspected daily for pregnancy and delivery. The day of birth was considered as postnatal day 0 (P0). Pups were sacrificed by decapitation. Animal cohorts were completely separated from one experiment to another and animals were not re-used from one experiment to another. Between tests, pups stayed with their dams and there was no cross-fostering before, during, or after the experiments. For all experiments, a total of 73 litters of pups were used. For a given experiment, P2 female and male pups (2.26–2.35 g) were randomly assigned to an experimental group, so that all experimental groups were represented in each litter and so that the female/male distribution was equal among groups.

### Determination of the Effective Dose of Remifentanil for Inducing Sedation in P2 Pups

Three doses (50, 250, or 500 ng/g) of the clinically used remifentanil formulation, Ultiva® (GlaxoSmithKline, Rueil-Malmaison, France), were tested to evaluate the effective dose for inducing sedation. P2 pups received three intraperitoneal (ip) injections of remifentanil (20 μl/g) over a 10-min period or the same volume of NaCl 0.9%. Sedation time was measured until 30 min after the last injection using the righting reflex test. The pups were placed supine on a flat surface and their mean time to return to a normal prone position during two consecutive trials was recorded (cut-off: 60 s). Behavioral test to measure sedation time was carried out during the first part of the light phase (between 9 and 11 a.m.).

### Determination of Plasma Remifentanil Concentration

Only P2 pups ip injected with 3 × 500 ng/g of remifentanil were used. Five minutes after the last injection, trunk blood from five pups was collected, and pooled in a heparinized tube. The tubes were immediately placed in an ice-water bath and then stored at −20°C. Remifentanil levels from 14 plasma pools (i.e., 70 pups) were quantified by combining reverse-phase high-performance liquid chromatography analysis with tandem mass spectrometry. After precipitating plasma in methanol, samples were centrifuged (14,000 rpm, 5 min) and supernatants were analyzed on a 0.21 × 50 mm Alltima HP C18 HL, 3 μm (Grace, Epernon, France) at a constant flow rate (200 μl/min). The mobile phase consisted of a gradient established over 5 min with 0.2% formic acid and 2 mM ammonium formate in acetonitrile. The concentration of remifentanil was determined using a calibration curve.

### Quantification of Physiological Parameters

P2 pups were ip injected with remifentanil (3 × 500 ng/g) or NaCl 0.9% as described above.

#### Ventilation and Heart Rates

The remifentanil (remi) and NaCl groups were compared to untreated pups. Animals were maintained in a cotton nest and filmed for 5 min. Two investigators blinded to the experimental group counted the number of breaths within 30 s, three times per pup. Heart rate was monitored for 5 min immediately after the last injection of remifentanil or NaCl. Electrocardiogram recordings were taken using subcutaneous surgical steel needle electrodes (29-gauge, 12-mm length) taped to the pup's foot pads. The signal was acquired at a 1,000-μs rate by using a PowerLab data acquisition system and biopotential amplifiers operated with LabChart 8.0 software (AD Instruments Ltd, Oxford, UK), and the data were analyzed using the electrocardiogram module of LabChart Pro 8.0 software.

#### Blood Gas Analysis

Pups were decapitated immediately after the last injection of remifentanil or NaCl, and trunk blood (venous and arterial blood) from seven pups was collected and pooled in heparinized capillary tubes. Blood gas (pCO_2_ and pO_2_) and pH were measured from eight blood pools using a blood gas analyzer (ABL800 Flex, Radiometer, Brea, CA, USA).

### Ibotenate Administration: Model of Excitotoxic Brain Lesions

Ibotenate (Tocris Bioscience, Bristol, UK) was diluted in 0.1 M phosphate buffered saline containing 0.02% acetic acid and intracortical (ic) injection was performed in P2 pups immediately after the last ip injection of remifentanil (500 ng/g; Ibo/remi group) or NaCl 0.9% (Ibo/NaCl group). Remifentanil administration preceded the ibotenate injection because our protocol aimed to mimic the conditions of general anesthesia before preterm cesarean delivery. In some experiments, a third group of unlesioned and untreated pups was added (unlesioned group). As previously described (20, 32), injections were given under a warming lamp using stereotaxic guidance with a 26-gauge needle mounted on a Hamilton syringe micro-dispenser device. The needle was inserted 2 mm under the external surface of the scalp skin in the frontoparietal area of the right hemisphere, 2 mm from the middle in the lateral-medial plane, and 2 mm anterior from the sagittal suture in the rostro-caudal plane. Two 1-μl boluses, each containing 5 μg of ibotenate, were injected 30 s apart. After the procedure, the pups returned to their dams. Pilot injection tests were performed with CellTracker Red (Invitrogen, Cergy-Pontoise, France) to standardize the injection protocol.

### Measurement of Brain ROS Production by Electron Paramagnetic Resonance Spectroscopy

ROS production was evaluated using electron paramagnetic resonance spectroscopy in the Ibo/NaCl and Ibo/remi groups at 10 min and 5 h postlesion during the increased ROS production period (33) and was compared to the unlesioned group. A frontal section encompassing the ibotenate injection site was dissected and an equivalent section was dissected from the brains of unlesioned pups. Tissues were frozen in liquid nitrogen and kept at −80°C until analysis. Tissues were homogenized (Polytron, Montreal, Canada) in Krebs/HEPES buffer (pH 7.4) and incubated at 37°C for 60 min in the same buffer containing the chelators deferoxamine (25 mM) and diethyldithiocarbamate (5 mM), and spin probe CMH (1-hydroxy-3-methocarbonyl-2,2,5,5-tetramethyl pyrrolidine hydrochloride; 500 mM; Noxygen, Hamburg, Germany). Then, samples were taken up into an insulin syringe, frozen in liquid nitrogen, and kept at −80°C until EPR measurement. Spectra of the oxidized product of CMH (CM°) were recorded at 77 K in a liquid nitrogen-cooled Dewar using an MS400 spectrometer (Magnettech, Berlin, Germany) with the following acquisition parameters: microwave power 20 mW, microwave frequency 9.5 GHz, modulation amplitude 5 G, modulation frequency 100 kHz, gain 500, team sweep time 60 s, for one scan. Intensity of the spectra was expressed in arbitrary units (AU) per milligram of protein.

### *In situ* Labeling and Quantification of Cortical Caspase Activity

Caspase activity was measured in frontal brain sections at 5 h postlesion using the CaspACE FITC-VAD-FMK *in situ* marker from Promega (Charbonnières les Bains, France). This fluorescent caspase inhibitor enters cells and is cleaved when activated caspases release the fluorescent FITC group. After sacrifice, brains were rapidly dissected to isolate the cerebral hemisphere. Meninges covering the brain were carefully removed and the brain was immediately placed into ice-cold artificial cerebrospinal fluid (aCSF) containing (in mM): NaCl, 125; KCl, 3; CaCl_2_, 2; NaH_2_PO_4_, 1.2; NaHCO_3_, 26; D-Glucose, 10; pH 7.4. Frontal sections (250-μm thick) were cut at 4°C using a vibratome VT1000S (Leica, France). Slices immediately adjacent to the site of the ic injection were collected and incubated with 10 μM CaspACE FITC-VAD-FMK for 20 min at 37°C in a humidified incubator under a controlled atmosphere of 5% CO_2_/95% air. Then, slices were washed 3 times with warm aCSF and fixed overnight in 4% paraformaldehyde (PFA) in phosphate buffered saline (PBS). Labeling was visualized with a Leica DMI 6000B microscope. A region of interest (ROI) was drawn in the neocortical layers around the site of injection. ROI were identified using the Atlas of Paxinos of the developing mouse brain. The same Bregma coordination was used among individuals to ensure reproducibility (2.91–3.51 mm). For each ROI, a threshold was set to distinguish the caspase-FITC-positive structures from the background, and the proportion of the labeled area was determined by image segmentation with Metamorph analysis software (Roper Scientific, Lisses, France).

### Fragmented DNA Detection by Enzyme-Immunoassay and Cleaved Caspase-3 Immunohistochemistry

The presence of fragmented DNA after induced cell death was assessed by measuring the cytoplasmic histone-associated DNA using a specific two-side ELISA with an anti-histone primary antibody and a secondary anti-DNA antibody, according to the manufacturer's instructions (Cell Death Detection ELISA^PLUS^, Roche Diagnostics, Mannheim, Germany). P2 pups from Ibo/NaCl and Ibo/remi groups were sacrificed at 24 h postlesion. At P3, brains from Ibo/NaCl, Ibo/remi, or unlesioned pups from the same litters were rapidly dissected. From each right hemisphere, a frontal section (2-mm thickness) from either side of the ibotenate injection site was dissected for use in the cell death detection ELISA assay. An equivalent section was dissected from brain of unlesioned pups. Brain tissue was homogenized in 1 ml of lysis buffer and incubated for 30 min at room temperature (21°C). After centrifugation to remove nuclei and cellular debris, the cytoplasmic fractions were diluted 1:2 (vol/vol) with lysis buffer. Then, 20 μl from each sample was transferred to a 96-well-plate precoated with an anti-histone antibody, to which 80 μl of immunoreagent mix was added. After incubation and washes, the wells were treated with the chromogen 2,2′-azinobis (3-ethylbinzothiazoline) sulfonic acid as a substrate. The color intensity was read at 405 nm, while that at 490 nm was used as a blank. The optical density of each sample was normalized to each sample's protein concentration.

The effect of remifentanil on the apoptotic process was illustrated at P3 by cleaved caspase-3 immunohistochemistry. Two frontal slices (250-μm thick) immediately adjacent to the site of the ic injection were cut as previously described. These brain slices were fixed with 4% PFA and incubated with rabbit antibodies against cleaved caspase-3 (#9661, Cell Signaling Technology, Boston, MA, USA) and diluted (1:200) in PBS containing 1% bovine serum albumin and 0.1% Triton X-100. Sections were then rinsed with PBS for 20 min and incubated with the same incubation buffer containing an Alexa Fluor® 488 donkey anti-rabbit IgG (Invitrogen). The specificity of the immunoreaction was controlled by substituting the primary antibodies with PBS.

### Multiplex Cytokine

At 24 h postlesion, brain cytokine analysis was performed using the Meso Scale Discovery (MSD) proinflammatory panel 1 mouseV-PLEX kit. This cytokine panel allows for the simultaneous measurement of IL-1β, IL-2, and TNF-α (K15048, MSD, Rockville, MD, USA). Samples were diluted (1:2) and assays were performed using the standard MSD protocol. The plate was read using a Sector Imager 2400 and data were analyzed using the MSD Discovery Workbench software v 4.0.

### GFAP Immunohistochemistry and Western Blot

The effect of remifentanil on reactive astrogliosis was evaluated at 24 h postlesion. Brains were immediately fixed in 4% formalin for 7 days and embedded in paraffin. Slices (7-μm thick) immediately adjacent to the site of the ic injection were cut on a microtome (Leica 2035 Biocut, Leica Microsystems, Wetzlar, Germany). Immunostaining of the astrocyte marker GFAP was performed using a goat anti-GFAP (sc-6170, dilution 1:200, Santa Cruz Biotechnology, Dallas, TX, USA) successively and an Alexa Fluor® 594 donkey anti-goat IgG (A-11058, dilution 1:400, Invitrogen, Carlsbad, CA, USA). The specificity of the immunoreaction was controlled by substituting the primary antibody with PBS. Four sections/animal were taken and an ROI (30,000 μm^2^) in the interior zone bordering the lateral ventricle was drawn and repeated from one slice to another. For each ROI, a threshold was set to distinguish the labeling from the background and the proportion of the labeled area was determined by image segmentation with Metamorph analysis software.

For Western blot analysis, the cortex from the ibotenate-injected hemisphere was rapidly dissected and total proteins were extracted using 250 μl lysis buffer containing 1% phosphatase inhibitor and 1% protease inhibitor. After centrifugation of the homogenates (15,000 × rpm, 4°C, 15 min), the supernatants were subjected to Western blotting. Protein extracts (100 μg) were suspended in Laemmli buffer (100 mM HEPES, pH 6.8, 10% β-mercaptoethanol, 20% SDS) and heated for 5 min. They were then loaded on 15% SDS-polyacrylamide gels and electroblotted onto nitrocellulose membranes. Commercial markers (Biorad) were used as molecular weight standards. Membranes were incubated in blocking solution (5% milk in Tris-buffered saline containing 0.1% Tween 20) at room temperature for 1 h, then overnight with goat anti-GFAP (sc-6170; 1:1,000, Santa Cruz Biotechnology, Dallas, TX, USA). After incubation with the corresponding peroxidase-conjugated secondary antibodies (donkey anti-goat, sc-2033, dilution 1:10,000, Santa Cruz Biotechnology), proteins were visualized using enhanced chemiluminescence ECL Plus immunoblotting (BioRad Laboratories, Marnes la Coquette, France). To verify equal protein loading, membranes were stripped and reprobed with mouse β-actin antibodies (A5441, 1:5,000, Sigma-Aldrich, St. Louis, MO, USA). The intensity of the immunoreactive bands was quantified using a blot analysis system (BioRad Laboratories) and data were expressed as GFAP/β-actin.

### Determination of Lesion Size

Five days after ic ibotenate injection, pups were sacrificed by decapitation. Brains were immediately fixed in 4% formalin for 7 days and embedded in paraffin. The size of cortical and white matter lesions can be defined by their extent along 3 orthogonal axes: the mediolateral axis (in the coronal plane), the radial axis (also in the coronal plane, from the pial surface to the lateral ventricle), and the fronto-occipital axis (in the sagittal plane). In previous studies using this model, an excellent correlation among measurements along the 3 axes was observed (21, 23, 34). Based on these findings, serial sections (7-μm thick) of the entire brain were cut in the coronal plane. Coronal sections were stained with Cresyl violet to determine the maximal extent of the lesion. The ibotenate-induced lesion is characterized by cortical neuronal depopulation and/or necrosis and/or the presence of cysts or atrophied white matter. The maximal fronto-occipital diameter of the lesion was determined as the number of sections multiplied by the thickness of one section (7 μm) and used as an index of lesion size. Two investigators blinded to the treatment groups independently determined the lesion size in each brain.

### Behavioral Assessments of Neonate Mice Sensorimotor Development

In a first set of experiments, the effect of remifentanil was evaluated in the lesional model and in a second set of experiments, the effect of remifentanil was evaluated in unlesioned pups. A third group of untreated P2 pups was included in both sets of experiments. Sensorimotor development was assessed by negative geotaxis and suspension tests (29–31, 35). The pups were weighed and tested between 9:00 and 11:00 a.m. Negative geotaxis, assessed from P6 to P8, is a postural reaction where the animal returns itself to an upright position when it is placed on an inclined surface with its head facing downward. The animals were placed on a 20° tilted plane with their head facing down, and the mean time to realize a full rotation of 180°, face up, was measured in each animal. The grasping reflex/grip strength was evaluated by the suspension test from P10 to P12. For the suspension test, a nylon thread was suspended 15 cm above a soft surface. Pups were gently held and their forepaws were brought into contact with the thread in order to provoke a grasping reflex. The mean time the pups were able to hold onto the nylon thread with their forepaws was recorded.

### Juvenile Mouse Behavior in the Open Field Test

P2 lesioned (Ibo/NaCl and Ibo/remi groups) and unlesioned (NaCl and remi groups) pups were observed for exploratory and locomotor activity at P18. All tests were performed between 9 a.m. and 1 p.m. Animals were isolated for 10 min in individual cages before being placed in the experimental device (40 × 40 × 30 cm), and the total distance traveled in an open field during a 30-min period was recorded using an automated image analysis system (Ethovision XT9.0, Noldus Information Technologies, Vageningen, The Netherlands). When mice were introduced in the open field, they were mainly inclined to explore the peripheral zone of the apparatus. This tendency to remain close to the wall, called thigmotaxis, is considered as an index of anxiety in rodents (36, 37). After a 5-min habituation period, thigmotaxis was assessed by the time spent in the central area of the open field (30 × 30 cm) during five successive 1-min periods. Four animals could be tested in parallel and male and female mice were tested separately.

### Statistical Analysis

Statistical analyses were performed using PRISM (Graphpad, San Diego, CA, USA). The data were plotted as the mean ± SEM. To evaluate the impact of remifentanil on brain lesion size, a target sample size of 56 pups, equally distributed (*n* = 28 in each group), was calculated, using the statistical software provided by BiostaTGV (https://biostatgv.sentiweb.fr), to detect a 30% reduction in the size of an ibotenate-induced lesion, with a type I error (two-sided) of 5% and a power of at least 90%. The other sample sizes were estimated based on previous experiments (19, 29, 30, 38, 39). A *p* < 0.05 was considered statistically significant. Tests used for each experiment, the number of independent experiments, and *p*-values are summarized in Supplementary Table 1, to homogenize the name of the supplementary data.

## Results

### Remifentanil Anesthesia, Pharmacokinetics, and Physiological Responses

**Figure 2:**
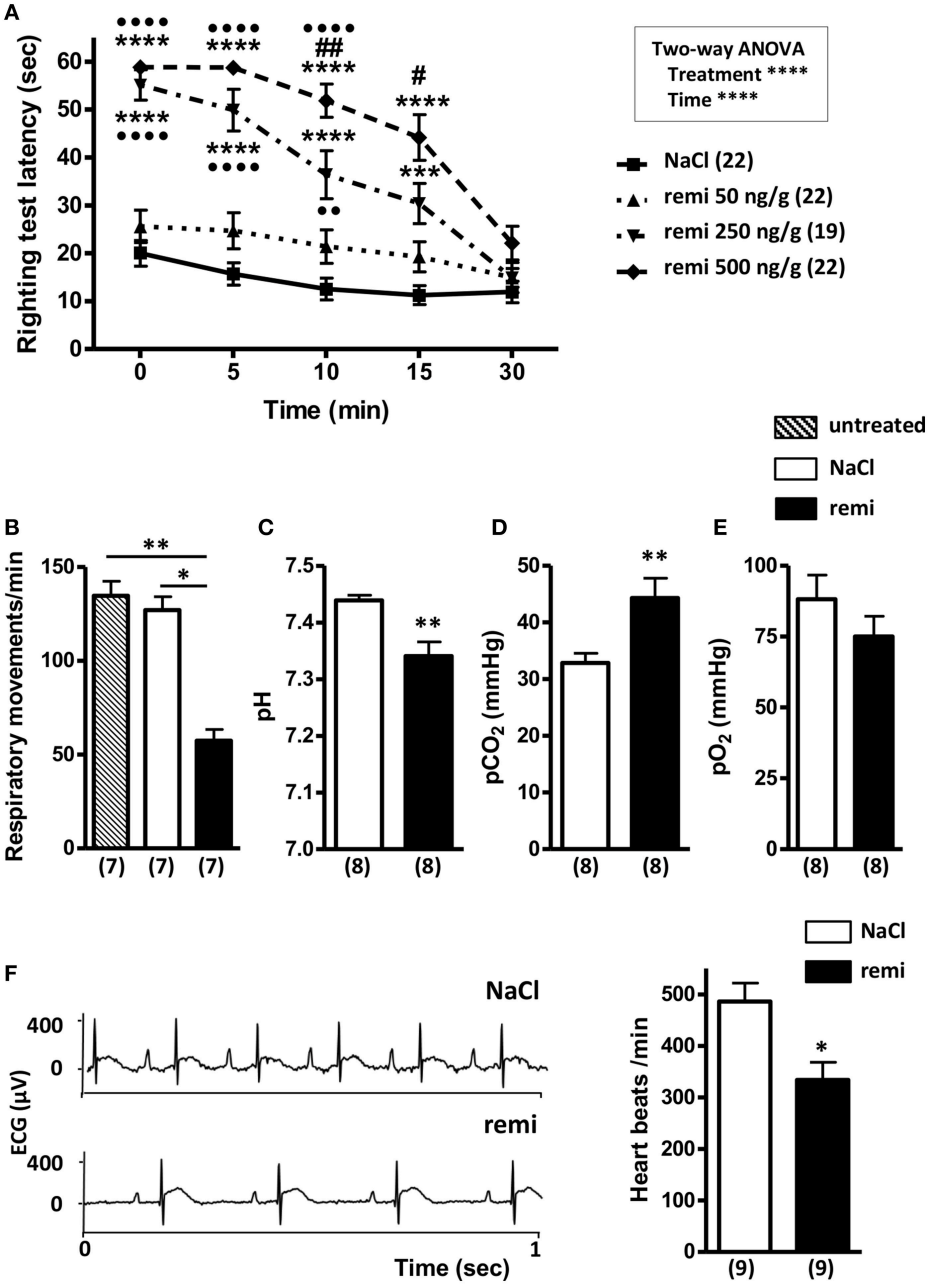
Effect of remifentanil exposure on postural righting reflex latency time and physiological parameters in P2 neonates. **(A)** Time differences needed to turn over and return to a prone position between remifentanil- and NaCl-treated postnatal day 2 (P2) mice. Pups received three injections of remifentanil (50, 250, or 500 ng/g) or NaCl 0.9% and each test was performed for 5 min. ^***^p < 0.001, ^****^p < 0.0001 vs. NaCl, ••*p* < 0.01, ••••*p* < 0.0001 vs. remifentanil (50 ng/g), #*p* < 0.05, ##*p* < 0.01 vs. remifentanil (250 ng/g) (two-way ANOVA, Bonferroni post-test). **(B)** Respiratory frequency evaluated during a 1-min period in untreated, remifentanil- (3 × 500 ng/g) and NaCl-treated P2 mice. ^**^p < 0.01 vs. untreated, ^*^p < 0.05 vs. NaCl (Kruskal-Wallis test). **(C–E)** Blood gas analyses (pH, pO_2_, and pCO_2_) in remifentanil- (3 × 500 ng/g) and NaCl-treated P2 mice. Values in parentheses represent the number of samples. Blood of 7 pups was pooled by sample. ^**^p < 0.01 (Mann-Whitney test). **(F)** Effect of remifentanil anesthesia on heart rate in P2 pups. Representative electrocardiogram tracing from P2 mice injected with remifentanil (3 × 500 ng/g) or saline (left panel). Quantification of heart beats recorded for 1 min in remifentanil and control groups (right panel) ^*^p < 0.05 (Mann-Whitney test). All values are expressed as the mean ± SEM. Number of animals or samples is indicated in parentheses.

Pups subjected to remifentanil administration at 250 and 500 ng/g spent significantly more time on their back than the control pups and those in the 50 ng/g group (*p* < 0.0001; Figure 2A). Compared to the control group, the pups injected with 50 ng/g of remifentanil exhibited only slight sleepiness, with no significant increase in latency times to return to a normal prone position. From 10 min post-injection, pups injected with 500 ng/g took significantly more time to turn over than pups injected with 250 ng/g remifentanil (*p* < 0.01 and *p* < 0.05 at 10 and 15 min, respectively), indicating a dose-dependent effect. At 30 min after remifentanil injection (250 or 500 ng/g), pups showed righting reflex latency times equivalent to those of NaCl-treated mice, indicating that animals recovered rapidly from anesthesia. There was no mortality during and after anesthesia. At 5 min after the last 500 ng/g injection, the plasma concentration of remifentanil was 10.03 ± 0.35 nmol/L (*n* = 14 pooled samples). Considering these results, the dose of 500 ng/g of remifentanil was subsequently used.

Remifentanil exposure led to a significant decrease of respiratory frequency compared to that observed in untreated and NaCl-treated pups (*p* < 0.01 and *p* < 0.05, respectively; Figure 2B). Blood gases measured following remifentanil administration revealed respiratory acidosis (*p* < 0.01; Figures 2C,D). Conversely, pO_2_ values did not significantly differ between remifentanil and NaCl-treated pups (Figure 2E). Without anesthesia, heart rate values were in an acceptable range (485.9 ± 36.1 bpm) (40). Remifentanil significantly reduced heart rates compared to that upon NaCl treatment (*p* < 0.05; Figure 2F).

### Remifentanil Prevented Ibotenate-Induced Brain Damage

**Figure 3:**
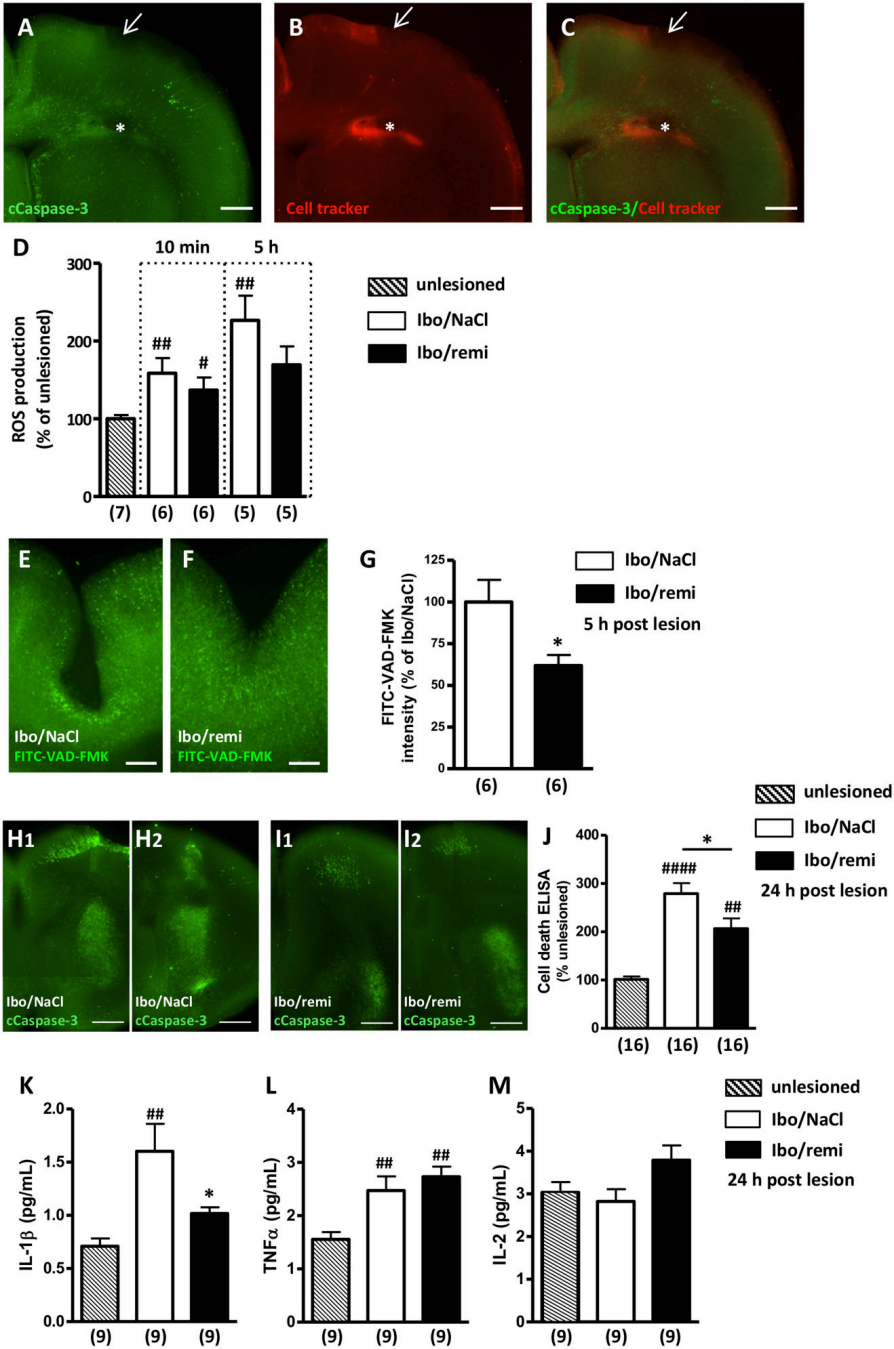
Effect of remifentanil exposure on ibotenate-induced ROS production, cell death and inflammatory cytokine levels in neonate mouse brains. **(A–C)** Photomicrographs illustrating the intracortical site injection at P2. **(A)** Visualization of cleaved caspase-3 immunoreactivity in P2 brains 5 h after intracortical injection of saline. Arrow indicates the injection site in the P2 cortex. **(B)** CellTracker Red (CT-red) was used to determine the depth of the injection site. Note the cavity induced by the injection (^*^). **(C)** Overlay of **(A,B)**. Scale bar equals 400 μm. **(D)** ROS production was assessed at 10 min and 5 h postlesion by electron paramagnetic resonance spectroscopy in brain ipsilateral hemispheres of Ibo/NaCl and Ibo/remi groups and compared to ROS production in the unlesioned group. ^##^*p* < 0.01, ^#^*p* < 0.05 vs. unlesioned group (Mann-Whitney test). **(E–G)** Effect of remifentanil exposure (3 × 500 ng/g) on *in situ* caspase activity in the P2 cortex 5 h after ic ibotenate injection. Visualization of the FITC-VAD-FMK signal in the cortex of **(E)** NaCl**-** or **(F)** remifentanil**-**treated P2 pups injected with ic ibotenate. Note a stronger FITC-VAD-FMK signal around the site of the injection in the cortex of Ibo/NaCl pups **(E)** compared to that in Ibo/remifentanil pups **(F)**. Scale bar equals 200 μm. **(G)** Remifentanil exposure significantly decreased *in situ* caspase activity (FITC-VAD-FMK signal) in ibotenate-injected pups (Ibo/remi). ^*^p < 0.05 vs. Ibo/NaCl group (Mann-Whitney test). **(H,I)** Cleaved-caspase-3 immunoreactivity visualized on P3 in two frontal successive brain sections from **(H1,H2)** NaCl**-** or **(I1,I2)** remifentanil**-**treated pups injected with ic ibotenate. Note that the apoptotic signal in the injected side of the brain was weaker in the remifentanil-treated pups. Scale bar equals 100 μm. **(J)** Quantitative analysis on P3 of DNA fragmentation in brain extracts from NaCl- or remifentanil-treated pups injected with ic ibotenate on P2 compared to that in unlesioned P3 pups. ^##^*p* < 0.01, ^####^*p* < 0.0001 vs. unlesioned group (Kruskal-Wallis test), ^*^p < 0.05 vs. Ibo/NaCl group (Mann-Whitney test). **(K–M)** Quantitative analysis of cytokine levels IL-1β **(K)**, TNFα **(L)**, and IL-2 **(M)** in the P3 cortex from NaCl- or remifentanil-treated pups injected with ic ibotenate on P2 compared to unlesioned P3 pups. ^##^*p* < 0.01 vs. unlesioned group, ^*^p < 0.05 vs. Ibo/NaCl (Kruskal-Wallis test). Values are expressed as the mean ± SEM. Number of animals is indicated in parentheses.

Cleaved caspase-3 immunohistochemistry and a CellTracker probe were used to visualize the ibotenate injection site (Figures 3A–C). Excessive ROS production was detected in brains from the Ibo/NaCl group compared to levels in the unlesioned group at 10 min (M10, *p* < 0.01) and 5 h (H5, *p* < 0.01) after lesion formation. Conversely, in the Ibo/remi group, a weak increase of ROS was measured at M10 (*p* < 0.05), and at H5, ROS production did not significantly differ from that of the unlesioned group (*p* = 0.0513; Figure 3D).

At H5, ic ibotenate injection induced a large increase in capsage activity around the lesion site (Figures 3E,F), which was significantly reduced by remifentanil treatment (*p* < 0.05; Figure 3G).

Cleaved caspase-3 immunohistochemistry performed at P3 showed that ic ibotenate injection induced a widespread apoptotic signal in the ipsilateral hemisphere (Figures 3H1,H2). The immunolabeling was particularly dense in the cortex around the injection site and in the striatum. Conversely, the apoptotic immunolabeling observed in the ibotenate-treated hemisphere of the remifentanil group appeared weaker (Figures 3I1,I2).

To confirm the inhibitory effect of remifentanil on apoptotic processes visualized at P3, DNA fragmentation was analyzed. Ic ibotenate injection potently increased cell death in the ipsilateral hemisphere compared to that in the unlesioned group (*p* < 0.0001; Figure 3J). Remifentanil significantly decreased ibotenate-induced DNA fragmentation in the injected hemisphere (*p* < 0.05; Figure 3J).

Inflammatory mediators were quantified 24 h after the injury. Ic ibotenate injection potently increased pro-inflammatory cytokine IL-1β production in the ipsilateral hemisphere compared to levels in the unlesioned group (*p* < 0.01; Figure 3K). In the Ibo/remi group, IL-1β production was significantly decreased compared to that in the Ibo/NaCl group (*p* < 0.05; Figure 3K). Production of the pro-inflammatory mediator TNFα was significantly increased in the Ibo/NaCl and Ibo/remi groups compared to that in the unlesioned group (*p* < 0.05 and *p* < 0.001, respectively; Figure 3L). No difference was observed between the Ibo/NaCl and Ibo/remi groups. Levels of the anti-inflammatory cytokine IL-2 did not differ between groups (Figure 3M).

**Figure 4:**
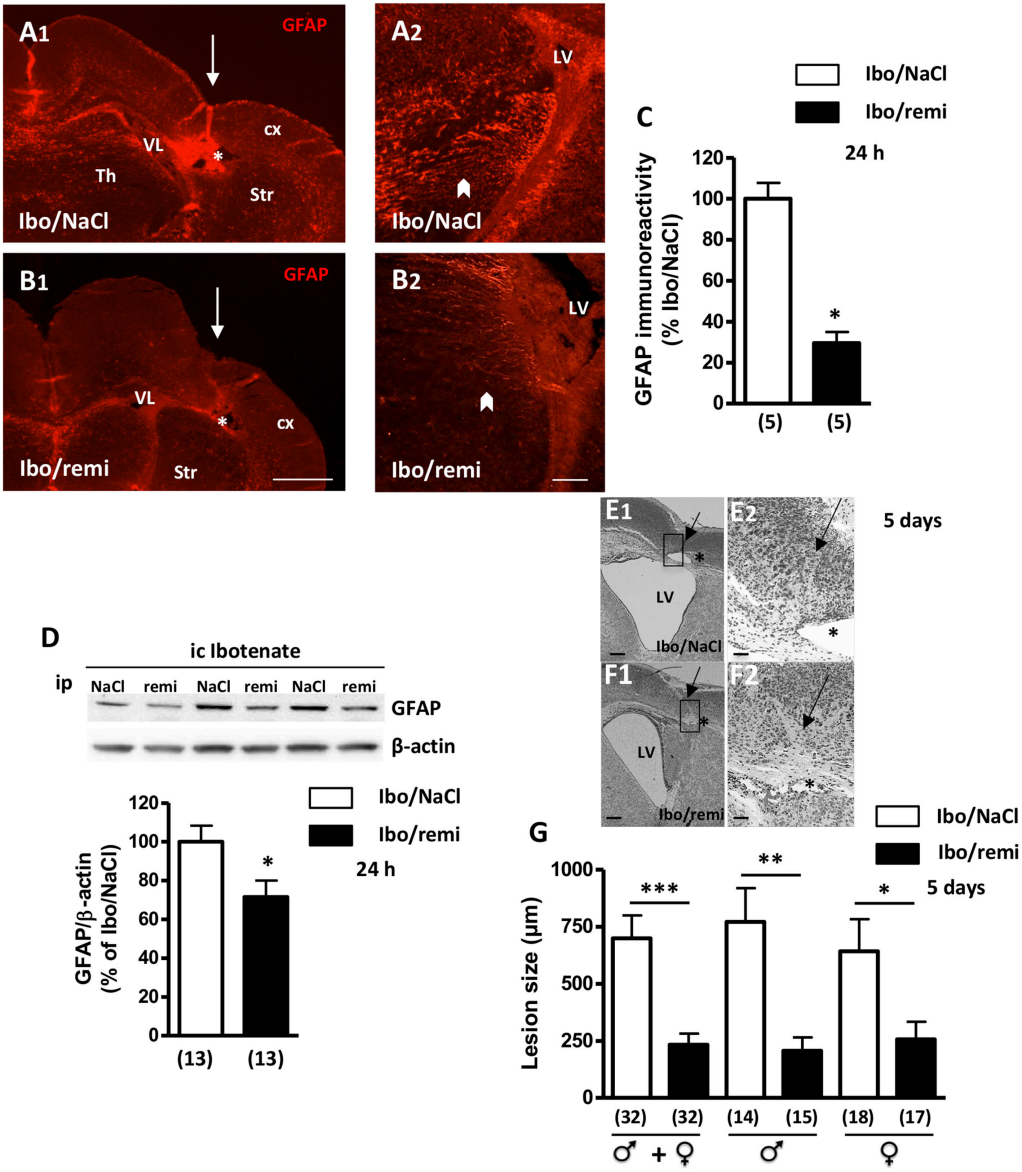
Effect of remifentanil exposure on reactive astrogliosis and lesion size induced by ibotenate injection. **(A,B)** GFAP immunolabeling performed at P3 in frontal brain sections from **(A1,A2)** NaCl- or **(B1,B2)** remifentanil-treated pups and injected with ic ibotenate at P2. In the ipsilateral hemisphere of a NaCl-treated pup (Ibo/NaCl), significant red GFAP immunostaining was visualized in the cortex (cx), thalamus (Th), striatum (Str), and areas bordering the lateral ventricle (LV) **(A1,A2)**. In the brain from a remifentanil-treated pup (Ibo/remi), the GFAP signal appeared weaker **(B1,B2)**. White arrows mark sites of ibotenate injection performed at P2. Asterisks indicate white matter cysts. In an Ibo/NaCl slice, blood autofluorescence occurred in the cyst. Arrowheads indicate GFAP immunolabeling observed under the lateral ventricle. Scale bars: 500 μm **(A1,B1)** and 50 μm **(A2,B2)**. **(C)** Quantitative analysis of GFAP immunostaining detected in an interior area bordering the lateral ventricle (illustrated in **A2,B2** by arrowheads). ^*^p < 0.05 vs. Ibo/NaCl group (Mann-Whitney test). **(D)** Quantification by Western blotting of GFAP protein expression in cortical extracts from P3 Ibo/NaCl and Ibo/remi pups. Representative immunoblots from cortical extracts are shown. Densitometric measurements were normalized to β-actin levels. Values are expressed as the mean ± SEM. ^*^p < 0.05 vs. Ibo/NaCl group (Mann–Whitney test). **(E,F)** Cresyl violet-stained sections showing brain lesions induced by ibotenate injected at P2 and studied at age P7, in Ibo/NaCl group **(E1,E2)** and Ibo/remi group **(F1,F2)**. Arrow points to the neuronal loss in cortical layers II–VI and star indicates the white matter lesion (cyst). The square, indicated in **E1,F1**, represents the region of interest visualized in **E2,F2**. LV, lateral ventricle. Scale bar: 200 μm **(E1,F1)** and 50 μm **(E2,F2)**. **(G)** Effect of remifentanil exposure on lesion size induced by ic ibotenate at P2 and studied at P7. The neuroprotective effect of remifentanil against neonatal excitotoxic damage was significant in both male and female pups. ^*^p < 0.05, ^**^p < 0.01, ^***^p < 0.001 vs. Ibo/NaCl group (Mann-Whitney test). Values represent the mean ± SEM. Number of animals is indicated in parentheses.

One day after the lesion was created, a decrease in cortical thickness was observed, and a destructive cyst of white matter was already present (Figure 4A1). GFAP immunodetection was used to examine astroglial reactivity. In the ipsilateral hemisphere to the lesion of an Ibo/NaCl pup, GFAP-positive astrocytes were found in the cortex, striatum, and thalamus, with the highest signal being observed in the interior zone bordering the lateral ventricle (Figures 4A1,A2). In the ipsilateral hemisphere of Ibo/remi pups, immunolabeling appeared much weaker than that in Ibo/NaCl pups (Figures 4B1,B2). Evaluation of the immunolabeled area in the interior zone bordering the lateral ventricle revealed a drastic decrease in astroglial reactivity in the Ibo/remi group (*p* < 0.05; Figure 4C). Western blot analysis showed a significant decrease in cortical GFAP levels after remifentanil treatment compared to the control levels (*p* < 0.05; Figure 4D).

After ic ibotenate and ip NaCl injection at P2, pups developed cortical lesion shrinkage of the white matter together with cyst formation at P7 (Figures 4E1,E2). Remifentanil treatment reduced the size of ibotenate-induced cortical lesions (up to 67%, *p* < 0.001; Figures 4F1,F2,G) in both males and females (*p* < 0.01 and *p* < 0.05, respectively; Figure 4G). Similarly, in the Ibo/remi group, the occurrence of cystic lesions in white matter was lower than that in the Ibo/NaCl group (51 vs. 62%; data not shown), but the difference was not statistically significant (Fisher's exact test, *p* = 0.4553).

At P7, the survival rate was similar between the Ibo/remi and Ibo/NaCl groups (Supplementary Table 2).

### Effect of Remifentanil Treatment on Sensorimotor Development of P2 Lesioned and Unlesioned Pups

**Figure 5:**
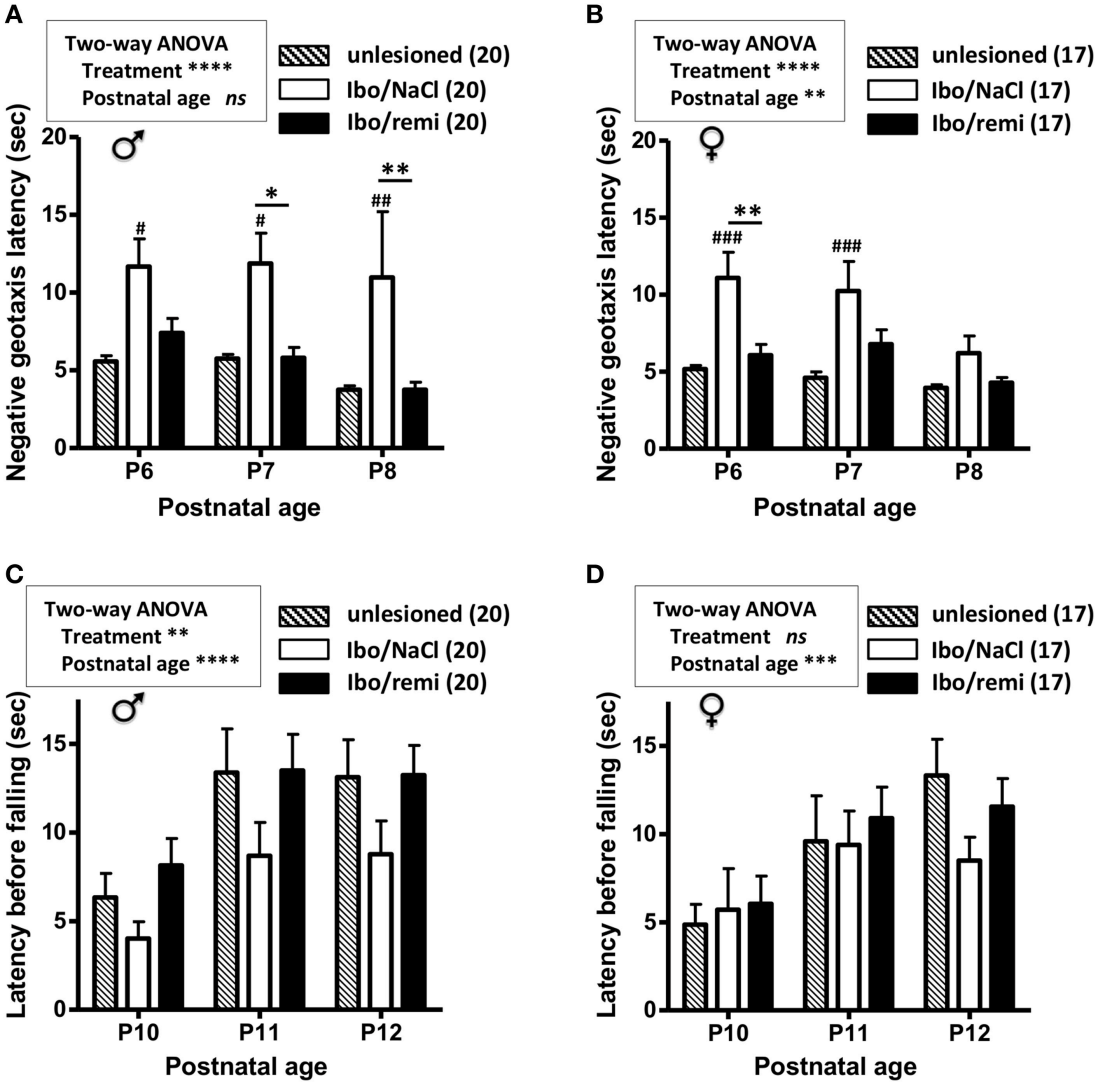
Effect of remifentanil exposure on sensorimotor performances of mice neonates injected with ibotenate at P2. **(A,B)** Quantification of negative geotaxis reflex in male **(A)** and female **(B)** neonates. Time necessary to rotate 180° was measured at P6 to P8 in unlesioned pups and ibotenate-injected pups exposed or not to remifentanil at P2. **(C,D)** Quantification of the grasping reflex in male **(C)** and female **(D)** neonates. Time latency before falling on the wire suspension test was measured at P10 to P12. Values are expressed as the mean ± SEM. ^*^p < 0.05, ^**^p < 0.01 Ibo/remi vs. Ibo/NaCl, #*p* < 0.05, ##*p* < 0.01, ###*p* < 0.001 Ibo/NaCl vs. untreated (two-way ANOVA test, Bonferroni post-test). Values represent the mean length of the lesions ± SEM. Number of animals is indicated in parentheses.

From P2 to P12, body weight was higher in the male Ibo/remi pups vs. that in the Ibo/NaCl pups (*p* < 0.0001; Supplementary Figure 1A). The body weight gain of female pups until P12 did not significantly differ between these groups (Supplementary Figure 1B). To determine if the neuroprotective effects of remifentanil positively affected sensorimotor development, negative geotaxis (P6-8) and grasping reflex (P10-12) tests were used. Male and female pups lesioned and exposed to remifentanil more rapidly rotated 180° than NaCl-treated pups (*p* < 0.0001) and showed performance close to that of the unlesioned pups (Figures 5A,B). The grasping reflex/grip strength analysis showed a higher latency before falling in male remifentanil-treated pups (*p* = 0.0054; Figure 5C), which exhibited a similar performance to that of unlesioned pups. Until P11, the unlesioned females showed poor grasping reflex performance (Figure 5D). At P12, the latency times before falling in unlesioned females were close to those of unlesioned males (Figures 5C,D). At this age, females lesioned and treated with remifentanil exhibited higher latency times than those observed in NaCl-treated females, although the effect of remifentanil did not reach statistical significance (Figure 5D).

**Figure 6:**
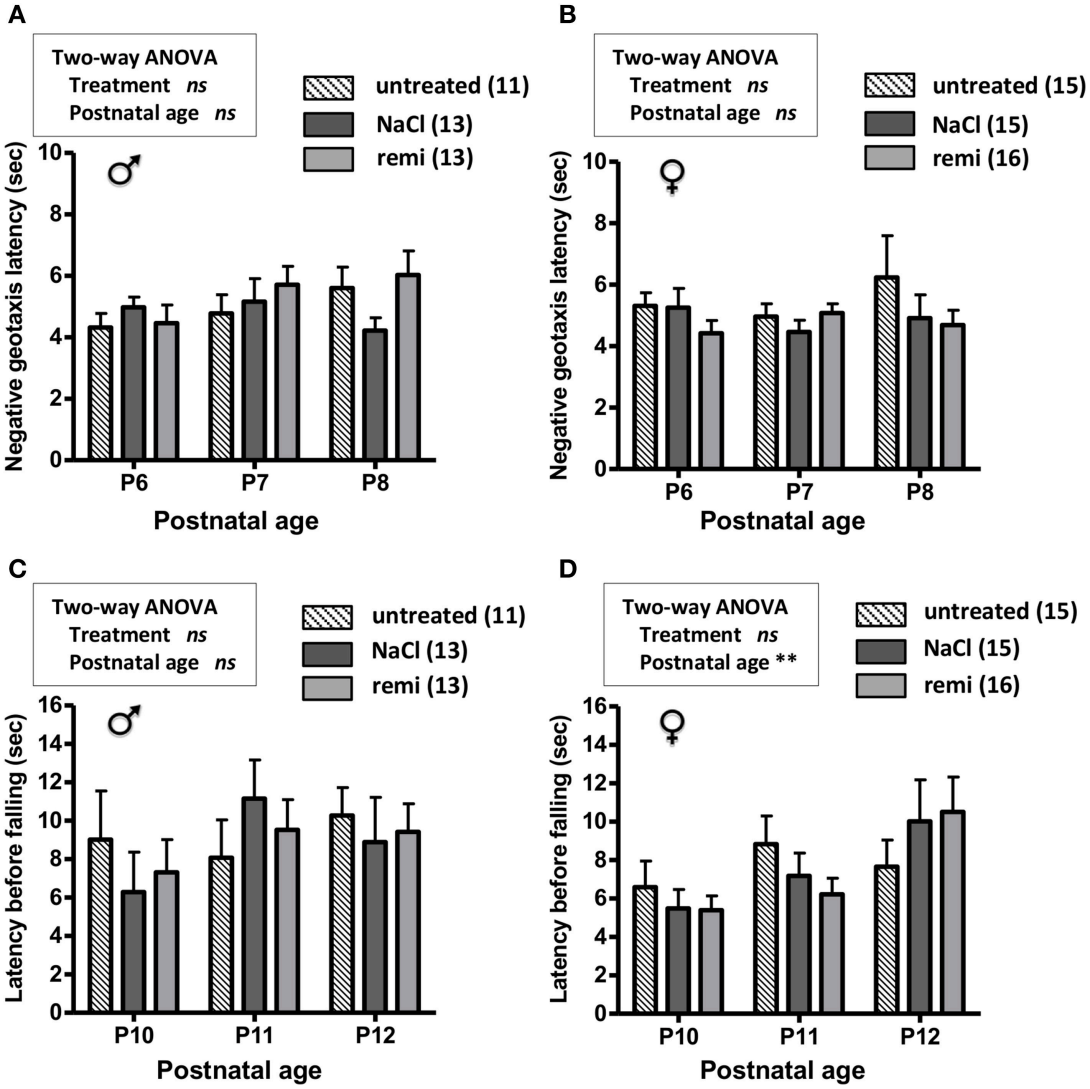
Effect of remifentanil exposure at P2 on sensorimotor performances of unlesioned mice neonates. **(A,B)** Quantification of negative geotaxis reflexes in male **(A)** and female **(B)** neonates. Time necessary to rotate 180° was measured at P6 to P8 in untreated pups and pups exposed or not to remifentanil at P2. **(C,D)** Quantification of the grasping reflex in male **(C)** and female **(D)** neonates. Time latency before falling on the wire suspension test was measured at P10 to P12. No differences were found (two-way ANOVA). Values are expressed as the mean ± SEM. Number of animals is indicated in parentheses.

The effect of remifentanil treatment was also investigated in unlesioned pups. Body weights were similar between the experimental groups from P2 and P12 (Supplementary Figures 1C,D). The measurements of negative geotaxis (P6-8; Figures 6A,B) and suspension (P10-12; Figures 6C,D) tests did not differ between the remifentanil and NaCl groups.

### Impact of Neonatal Remifentanil Exposure on the Locomotor Activity in Young Mice

**Figure 7:**
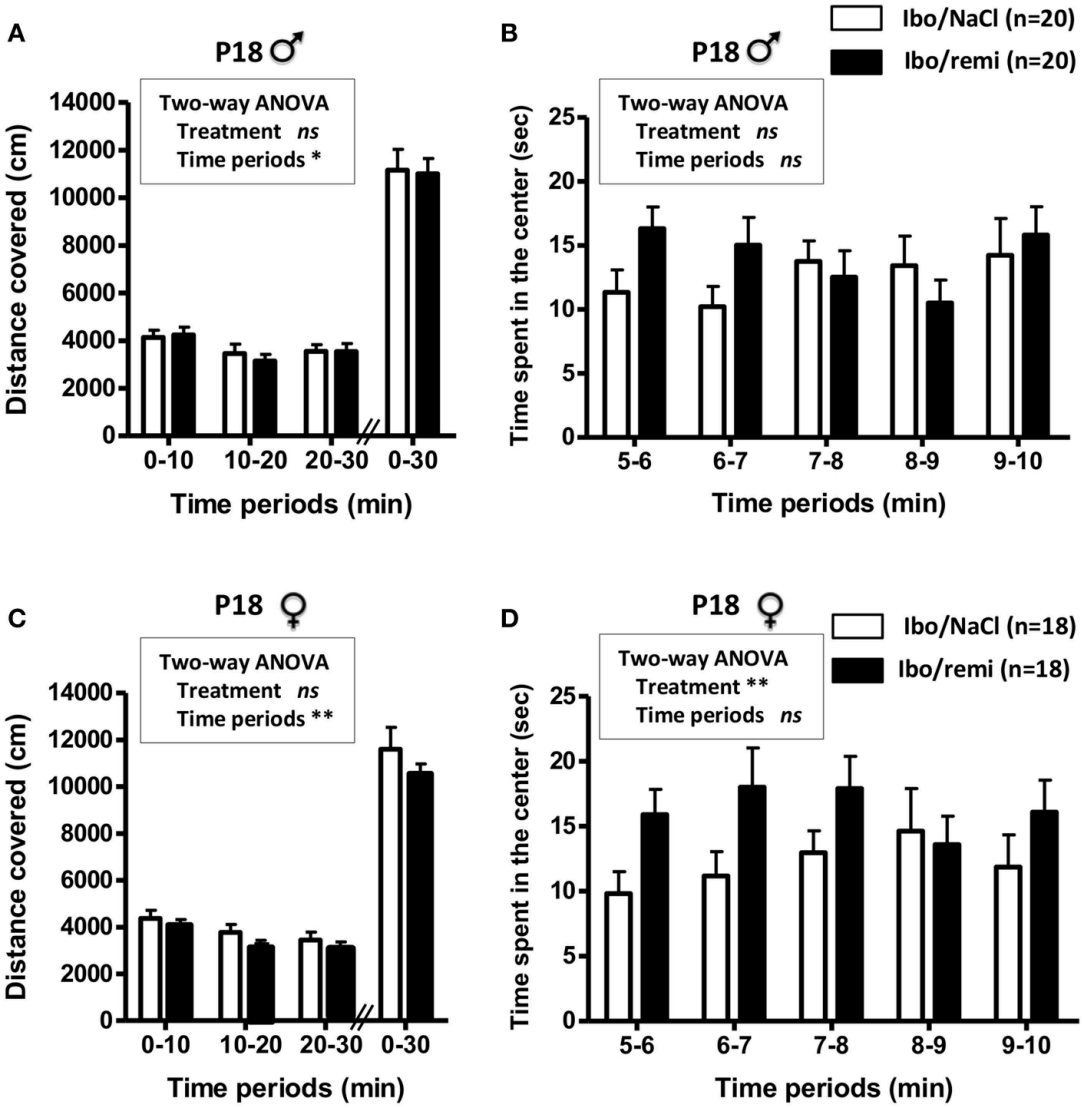
Effect of remifentanil exposure in the neonatal period on spontaneous motor activity of P18 juvenile mice lesioned with ibotenate at P2. **(A,B)** Quantification over 30 min of the total distance covered in the entire compartment **(A,C)** and the time spent in the center zone **(B,D)** by males **(A,B)** and females **(C,D)**. Animals were ic injected with ibotenate and exposed or not to remifentanil at P2 and studied at P18. Displacements were measured for three consecutive 10-min periods. The time spent in the center was measured for the first five consecutive 1-min periods. Experimental groups were compared using a two-way ANOVA. Values are expressed as the mean ± SEM. Number of animals is indicated in parentheses.

An open field test was conducted to assess locomotor activity before weaning (P18). Body weight did not differ between the Ibo/remi and Ibo/NaCl groups, in both males (8.06 ± 0.19 g vs. 8.01 ± 0.27 g, respectively) and females (7.78 ± 0.33 g vs. 7.62 ± 0.30 g, respectively). In males, no significant difference was found between the Ibo/remi and Ibo/NaCl groups regarding the distance covered during 30 min in the entire compartment (Figure 7A) or the time spent in the center during the first 5 min after the habituation period (Figure 7B). Although lesioned females injected with remifentanil at P2 covered a distance similar to that of NaCl-injected females in the entire compartment (Figure 7C), they spent significantly more time in the center than controls did (*p* = 0.0054; Figure 7D), suggesting that lesioned females exposed to remifentanil were less anxious than NaCl-treated mice. In another set of experiments, locomotor activity was measured in unlesioned mice. The distance covered in the entire open field during 30 min and the time spent in the center zone did not differ between remifentanil- and NaCl-treated animals regardless of sex (Supplementary Figure 2).

There were no deaths nor abnormal behavior between the end of the tests at P18 and P45. At P45, the body weight of P2 lesioned mice did not differ between the groups treated with remifentanil and saline, in males (33.45 ± 0.55 g vs. 32.69 ± 0.57 g, respectively) and females (24.96 ± 0.43 g vs. 25.00 ± 0.55 g, respectively).

## Discussion

This study provides experimental support highlighting the absence of a neurotoxic effect of remifentanil preconditioning under excitotoxic conditions in neonate mice, and even suggests a potentially neuroprotective effect. Indeed, remifentanil treatment decreased ROS production, cell death, and astrogliosis induced by ic ibotenate injection at P2, and led to a reduction in brain lesion size. In addition, remifentanil positively affected some sensorimotor tests of the lesioned pups, as performance improved in the negative geotaxis test for both males and females between P6 and P8, and in the grip test for males between P10 and P12. In the longer term, juvenile lesioned P18 females exposed to remifentanil spent significantly more time in the center of the open field than controls, suggesting that remifentanil-exposed juvenile females may be less anxious than control females.

Dose determination for an animal experimental study is a classical point of discussion. Thus, in our experiments, we first characterized the effective dose of remifentanil for inducing transient sedation in P2 neonates. The 500 ng/g dose in 3 injections appeared to give the most relevant clinical effect, although it may appear high compared to the doses usually used in humans (in humans, dosing ranges from 0.1 to 9 μg/kg/min or 1 to 5 μg/kg for intravenous bolus) (41, 42). However, this dose seems clinically appropriate as demonstrated by the righting reflex test and the plasma levels of remifentanil (10 nmol/L) in the pups, which were similar to human concentrations (from 10 to 100 nmol/L) (43). Drug metabolism and pharmacokinetics differ considerably between humans and mice, and discrepancies between effective doses are commonly observed. For example, ketamine and midazolam doses required to induce anesthesia in mice neonates are 10–30 times higher than the human doses (38, 44).

Cardiorespiratory consequences of remifentanil could have a specific impact on brain development. Bradypnea, bradycardia, and hypotension are common clinical side effects observed upon remifentanil treatment in human children (45). The significant hypercapnia observed in the remifentanil group, as a consequence of bradypnea, may have a specific effect on brain lesions. Indeed, hypercapnia might be associated with neurological disorders in human preterm neonates (46), and it causes cell damage and microvascular degeneration in immature rodent brains (47). It was not possible to assess the blood pressure in P2 pups given their low weight (about 2 grams); therefore, the hemodynamic effect of remifentanil was only evaluated by measuring the heart rate. As bradycardia in humans is associated with arterial hypotension (45), we can assume that pups treated with remifentanil, which exhibited a decreased heart rate, were probably hypotensive. In preterm infants, hypotension is associated with increased mortality, intraventricular hemorrhage, periventricular leukomalacia, and neurodevelopmental morbidity (48, 49). Taken together, the cardiorespiratory effects associated with remifentanil may, at worst, have a deleterious effect on ibotenate-induced brain damage.

Following ibotenate injection, the neonatal brain undergoes a series of cellular and molecular impairments ranging from inflammatory changes such as microglial activation as well as ROS and cytokine production within a few hours of the insult to neuronal loss within a few days, mimicking some aspects of the developmental lesions observed in human preterm neonates (23, 50). Our data showed that within the first hours postlesion, remifentanil treatment decreased ROS production and caspase-3 activity in the lesioned hemisphere of mouse pups. At 1 day postlesion, a weaker caspase-3 immunoreactive signal was seen and a decrease in cell death was observed in brains from remifentanil-treated pups, leading to a significant reduction of the lesion size at P7 (up to 67%). Altogether, our data suggested that remifentanil preconditioning is not harmful and may even reduce the severity of neonatal excitotoxic brain injury, probably involving an anti-apoptotic mechanism, as we previously showed *ex vivo* (19). Our results were consistent with those of previous studies that showed a neuroprotective effect of remifentanil against transient focal brain ischemia in adult rodents involving inhibition of neuronal apoptosis (51, 52). In the same way, a recent work demonstrated that remifentanil reduces isoflurane-induced apoptosis in the neonate rat brain (53).

The mechanisms underlying the potential neuroprotective action of remifentanil have been poorly investigated. In a previous *ex vivo* study using a model of acute cultured P2 brain slices, we first demonstrated that remifentanil was ineffective against necrotic death, whereas it significantly reduced caspase-3 activity and cortical cleaved caspase-3 levels (19). We showed that this anti-apoptotic effect involved the opioid and NMDA receptors (action is reversed by the opioid receptor antagonist naloxone and the NMDA antagonist MK801), and the mitochondrial-dependent apoptotic pathway (remifentanil inhibits cortical Bax protein expression and caspase-9 activity whereas it had no effect on caspase-8 activity). This anti-apoptotic effect may affect neurological development, as neuroapoptosis is necessary for proper brain development (54). However, the absence of deleterious effects of remifentanil on the sensorimotor performance of unlesioned mice in our experiments suggests that remifentanil did not negatively impact neurodevelopment.

Inflammation is increasingly recognized as a critical contributor to perinatal brain injury (55). During an excitotoxic challenge, leucocyte infiltration, microglial activation, and reactive astrogliosis participate in brain inflammation, triggering the release of many neurotoxic compounds such as free radicals and cytokines, which aggravate brain lesions (20, 56). We found that remifentanil alleviates brain ROS production and astrogliosis induced by ibotenate, suggesting that this opioid can modulate neuroinflammation. Moreover, while ibotenate induced inflammation with an increase of some pro-inflammatory cytokines (IL-1β and TNFα), remifentanil partially antagonized this effect by normalizing IL-1β production. In adult rats, remifentanil preconditioning has been shown to reduce brain damage from cerebral ischemia reperfusion and to decrease TNF-α expression, ROS production, and caspase-3 and -9 activities (57). In addition, in an inflammatory state, remifentanil suppressed an increase in IL-6 mRNA levels in mouse brains (58). Furthermore, *in vitro* data have demonstrated that remifentanil inhibits neutrophil migration and cytokine production by human activated neutrophils (59). All these data support an anti-inflammatory effect of remifentanil in neonatal brain lesions, which could participate in the beneficial impact of this opioid agent.

We also investigated whether remifentanil exposure could have a functional effect until P18, and we demonstrated that under lesional conditions, remifentanil counterbalanced some of the behavioral deficits observed through behavioral tests assessing motor activity and sensory development. We also demonstrated that the size of the ibotenate-induced cortical-subcortical lesion was significantly decreased in mice treated with remifentanil. In rodents, hypomyelination of the subcortical white matter may partly lead to delayed sensorimotor reflexes (60–62). Together, these data suggest that remifentanil could protect cortical structure and white matter, and thus, could preserve motor and proprioceptive integrative centers. Similarly, the geotaxis test results supported a functional link between the sensorimotor cortex and thalamic nuclei that integrate the vestibular system information being preserved, thus allowing the perception of rotation and vertical orientation.

In the grip test, the effect of the lesion was only detectable at P12 in females, but it did not reach statistical significance. Therefore, this behavioral test does not seem appropriate to estimate putative remifentanil protection in females. The open field test performed at P18 did not reveal motor deficits in the injured mice of either sex, whether treated with remifentanil or not. This absence of a deficit may be explained by the very strong cerebral plasticity in young rodents. Indeed, the first postnatal weeks are important for synaptogenesis and increased synaptic activity (63), leading to long-term functional recovery and compensation for motor impairment induced by a cerebral injury (64–66). We suggest that this compensatory mechanism is more apparent at the time of the open field test (performed at P18) than at the time of the negative geotaxis and grip tests conducted at earlier stages of development (performed between P6 and P12). In addition, because mice are placed in a new environment in the open field test, this test is also indicative of anxiety, which can be evaluated by the time spent in the center of the device in the first minutes of the test (36, 37). Our results showed that lesioned females treated with remifentanil spent more time in the central area than injured animals that received NaCl, suggesting a less anxious state of remifentanil-treated female animals. This anxiolytic effect may be related to the action of remifentanil on opiate receptors, the activation of which has been implicated in regulating emotional states, including anxiety (67).

## Conclusions

The present study provided the first evidence that the anti-apoptotic effects of remifentanil on the developing mouse brain previously shown *ex vivo* were also detectable *in vivo*, in a neonatal model of excitotoxic lesions. This anti-apoptotic effect was associated with diminished excitotoxicity (inflammation, astrogliosis, ROS production), reduced ibotenate-induced brain lesion size, and the prevention of some behavioral deficits occurring during the first days of life. Further experiments are needed to confirm if these findings carry into adulthood and to explore the mechanisms involved in this neuroprotective effect.

## Ethics Statement

This study was carried out in accordance with the Animal Research: Reporting *in vivo* Experiments (ARRIVE) guidelines. The protocol was approved by the French National legislation (ethical approvals no. 01316.02 and 01680.02).

## Author Contributions

CC designed the study, performed the experiments, collected the data, analyzed the data, and wrote the manuscript. MLec, IR-J, J-CD, LA-D, PC, BD, SM, BG, SJ, and FT helped design the study. MLec, MLeu, YR, SJ, and FT helped perform the experiments. IR-J and MLec performed the ROS experiments. PC performed the pharmacological tests. MLec, MLeu, YR, SJ, and FT helped collect the data. MLec, J-CD, LA-D, VR, SM, BG, SJ, and FT helped analyze the data. MLec, SM, BG, SJ, and FT helped write the manuscript. MLeu, IR-J, J-CD, LA-D, YR, VR, PC, and BD revised the manuscript. All authors have read and approved the final manuscript and are accountable for all aspects of the work in ensuring that questions related to the accuracy or integrity of any part of the work are appropriately investigated and resolved.

### Conflict of Interest Statement

The authors declare that the research was conducted in the absence of any commercial or financial relationships that could be construed as a potential conflict of interest.

## Supplementary Material

Supplementary Figure 1 Weight gain in male and female pups from P2 to P12. **(A,B)** Weight intake in male **(A)** and female **(B)** pups lesioned with ibotenate at P2 and injected with ip NaCl (Ibo/NaCl) or remifentanil (Ibo/remi). **(C,D)** Weight intake in unlesioned male **(C)** and female **(D)** pups injected at P2 with NaCl or remifentanil. Comparison of experimental groups was performed using a two-way ANOVA. Values are expressed as the mean ± SEM. Number of animals is indicated in parentheses.

Supplementary Figure 2 Effect of remifentanil exposure in neonatal period on spontaneous motor activity of P18 juvenile unlesioned mice. **(A,B)** Quantification over 30 min of the total distance covered in the entire compartment **(A,C)** and the time spent in the center zone by **(B,D)** males **(A,B)**, and females **(C,D)**. Animals were injected with remifentanil or NaCl at P2 and studied at P18. Displacements were measured for three consecutive 10-min periods. The time spent in the center was measured for the first five consecutive 1-min periods. No differences were found (two-way ANOVA). Number of animals is indicated in parentheses.

Supplementary Table 1 Statistical analysis.

Supplementary Table 2 Survival rates of NaCl- and remifentanil-treated pups injected at P2 with ibotenate at 5 days post-lesion.
