# Supplementary figures and images for: Beneficial Effects of Remifentanil Against Excitotoxic Brain Damage in Newborn Mice
Source: Front Neurol. 2019 Apr 24;10:407. doi: 10.3389/fneur.2019.00407 (PMC6491788; doi:10.3389/fneur.2019.00407)

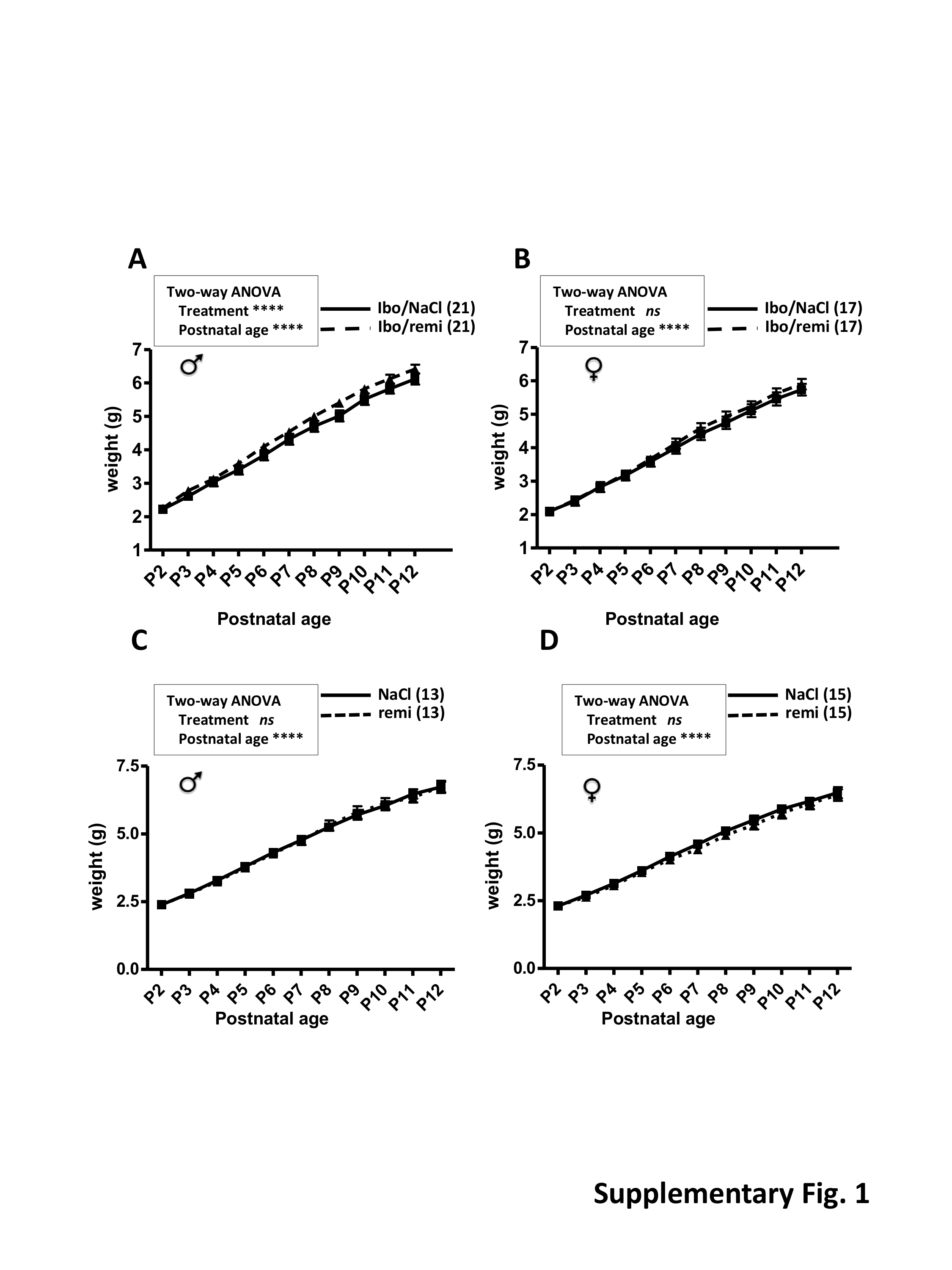

Supplement: Supplementary Figure 1 — Weight gain in male and female pups from P2 to P12. (A,B) Weight intake in male (A) and female (B) pups lesioned with ibotenate at P2 and injected with ip NaCl (Ibo/NaCl) or remifentanil (Ibo/remi). (C,D) Weight intake in unlesioned male (C) and female (D) pups injected at P2 with NaCl or remifentanil. Comparison of experimental groups was performed using a two-way ANOVA. Values are expressed as the mean ± SEM. Number of animals is indicated in parentheses. [file Image_1.TIFF]

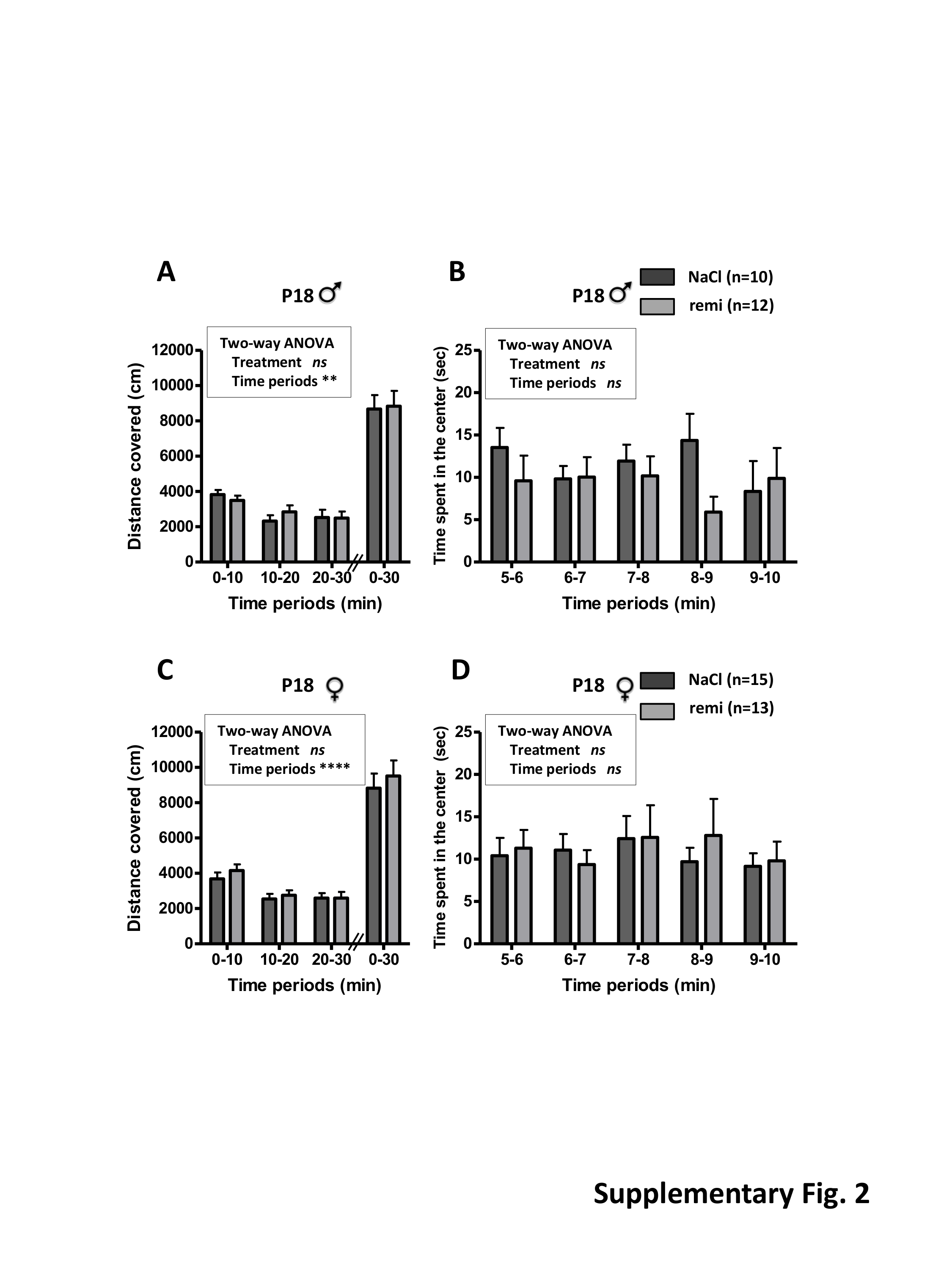

Supplement: Supplementary Figure 2 — Effect of remifentanil exposure in neonatal period on spontaneous motor activity of P18 juvenile unlesioned mice. (A,B) Quantification over 30 min of the total distance covered in the entire compartment (A,C) and the time spent in the center zone by (B,D) males (A,B), and females (C,D). Animals were injected with remifentanil or NaCl at P2 and studied at P18. Displacements were measured for three consecutive 10-min periods. The time spent in the center was measured for the first five consecutive 1-min periods. No differences were found (two-way ANOVA). Number of animals is indicated in parentheses. [file Image_2.TIFF]
