# Supplementary material for: Beneficial Effects of Remifentanil Against Excitotoxic Brain Damage in Newborn Mice
Source: Front Neurol. 2019 Apr 24;10:407. doi: 10.3389/fneur.2019.00407 (PMC6491788; doi:10.3389/fneur.2019.00407)
Supplement: Supplementary Table 1 — Statistical analysis. [file Table_1.DOCX]

**Supplemental Table 1:** Statistical analysis.

| **Cohort experiment** | **Experiments** | **Test** | **n**  **number of animals or samples** | **p value**  ***p<0.05; **p<0.01; ***p<0.001, ****p<0.0001** |
| --- | --- | --- | --- | --- |
| 1  (7 litters) | Righting reflex latency time  (Fig. 2A) | Two-way ANOVA, Bonferroni post-test | NaCl n= 22  remi 50 ng/g n= 22  remi 250 ng/g n= 19  remi 500 ng/g n= 22 | **Two-way ANOVA [F(DFn, DFd)]**  Interaction F(12, 405)= 5.167 p< 0.0001  Treatment factor F(3, 405)= 109.3 p< 0.0001  Time factor F(4, 405)= 35.2 p< 0.0001  **At 0 min**  remi 500 ng/g *vs* NaCl p< 0.0001 ****  remi 250 ng/g *vs* NaCl p< 0.0001 ****  remi 500 ng/g *vs* remi 50 ng/g p< 0.0001 ••••  remi 250 ng/g *vs* remi 50 ng/g p< 0.0001 ••••  **At 5 min**  remi 500 ng/g *vs* NaCl p< 0.0001 ****  remi 250 ng/g *vs* NaCl p< 0.0001 ****  remi 500 ng/g *vs* remi 50 ng/g p< 0.0001 ••••  remi 250 ng/g *vs* remi 50 ng/g p< 0.0001 ••••  **At 10 min**  remi 500 ng/g *vs* NaCl p< 0.0001 ****  remi 250 ng/g *vs* NaCl p< 0.0001 ****  remi 500 ng/g *vs* remi 50 ng/g p< 0.0001 ••••  remi 250 ng/g *vs* remi 50 ng/g  p= 0.0074 ••  remi 500 ng/g *vs* remi 250 ng/g  p= 0.0053 ##  **At 15 min**  remi 500 ng/g *vs* NaCl p< 0.0001 ****  remi 250 ng/g *vs* NaCl p= 0.0002 ***  remi 500 ng/g *vs* remi 250 ng/g  p= 0.0181 # |
| 2  (3 litters) | Respiratory movements  (Fig. 2B) | Kruskall-Wallis test | Untreated n= 7,  remi n= 7  NaCl n= 7 | Kruskal-Wallis statistic = 13.78  remi *vs* untreated p= 0.0015 **  remi *vs* NaCl p= 0.0134 * |
|  | Heart rate  (Fig. 2F) | Mann Whitney test | remi n= 9  NaCl n= 9 | Mann-Whitney U= 13.00 p= 0.0142 * |
| 3  (6 litters) | Blood gas analysis  (Fig. 2C-D-E) | Mann Whitney test | remi n= 8  NaCl n= 8  (Values represent the number of pooled samples. Blood of 7 pups was pooled by sample) | Fig. 1C Mann-Whitney U= 6.00 p= 0.0047 **  Fig 1D Mann-Whitney U= 6.00 p= 0.0074 ** |
| 4  (3 litters) | ROS production  (Fig. 3D) | Mann Whitney test | Unlesioned n= 7  At 10 min:  Ibo/NaCl, Ibo/remi n= 6  At 5 hours:  Ibo/NaCl, Ibo/remi n= 5 | **At 10 min**  Ibo/NaCl *vs* unlesioned Mann-Whitney U= 2.00 p= 0.0047 ##  Ibo/remi *vs* unlesioned Mann-Whitney U= 4.00 p= 0.0303 #  **At 5 hours**  Ibo/NaCl *vs* unlesioned Mann-Whitney U= 0.00 p= 0.0025 ## |
| 5  (3 litters) | *in situ* caspase activity  (Fig. 3G) | Mann Whitney test | Ibo/ NaCl n=6  Ibo/ remi n=6 | Mann-Whitney U= 5.00 p= 0.0411 * |
| 6  (4 litters) | DNA fragmentation  (Fig. 3J) | Mann Whitney test and Kuskall-Wallis test | Unlesionned n=16  Ibo/ NaCl n=16  Ibo/ remi n=16 | **Mann-Whitney** U= 67.00  Ibo/remi *vs* Ibo/NaCl p= 0.0275 *  **Kruskall-Wallis** statistic = 29.14  Ibo/NaCl *vs* unlesioned p< 0.0001 ####  Ibo/remi *vs* unlesioned p= 0.0014 ## |
| 7  (3 litters) | Inflammatory cytokines  (Fig. 3K-L-M) | Kruskall-Wallis test | Unlesionned n=9  Ibo/ NaCl n=9  Ibo/ remi n=9 | **Interleukin 1 beta**  Kruskall-Wallis statistic = 9.570  Unlesioned *vs* Ibo/NaCl p= 0.0017 ##  Unlesioned *vs* Ibo/remi p= 0.1834  Ibo/NaCl *vs* Ibo/remi p= 0.0307 *  **TNF alpha**  Kruskall-Wallis statistic = 14.18  Unlesioned *vs* Ibo/NaCl p= 0.0084 ##  Unlesioned *vs* Ibo/remi p= 0.014 ##  Ibo/NaCl *vs* Ibo/remi p= 0.3780  **Interleukin 2**  Kruskall-Wallis statistic = 4.542  Unlesioned *vs* Ibo/NaCl p= 0.6114  Unlesioned *vs* Ibo/remi p= 0.1575  Ibo/NaCl *vs* Ibo/remi p= 0.0833 |
| 8  (3 litters) | Quantitative analysis of GFAP  (Fig. 4C) | Mann Whitney test | Ibo/ NaCl n=5  Ibo/ remi n=5 | Mann-Whitney U= 0.00 p= 0.0159 * |
| 9  (4 litters) | WB GFAP  (Fig. 4D) | Mann Whitney test | Ibo/ NaCl n=13  Ibo/ remi n=13 | Mann-Whitney U= 43.00 p= 0.0355 * |
| 10  (8 litters) | Size lesion induced by ibotenate  (Fig. 4G) | Mann Whitney test | Ibo/NaCl n=32  Ibo/NaCl: ♂=14; ♀=18  Ibo/remi n=32  Ibo/remi: ♂=15; ♀=17 | **Ibo/remi *vs* Ibo/NaCl (both gender)**  Mann-Whitney U= 250.5  p= 0.0003 ***  **Ibo/remi *vs* Ibo/NaCl (♂)**  Mann-Whitney U= 34.00  p= 0.0018 **  **Ibo/remi *vs* Ibo/NaCl (♀)**  Mann-Whitney U= 92.50  p= 0.0420 * |
| 11  (7 litters) | Negative geotaxis of male mice with ibotenate  (Fig. 5A) | Two-way ANOVA, Bonferroni post-test | Unlesionned n=20  Ibo/ NaCl n=20  Ibo/ remi n=20 | **Two-way ANOVA [F(DFn, DFd)]**  Interaction F(4, 176)= 0.2235 p= 0.9250  Treatment factor F(2, 176)= 13.21 p< 0.0001  Postnatal age factor F(2, 176)= 1.253 p= 0.2881  **P6**  Ibo/NaCl *vs* unlesioned p= 0.0405 #  **P7**  Ibo/NaCl *vs* unlesioned p= 0.0369 #  Ibo/remi *vs* Ibo/NaCl p= 0.0335 *  **P8**  Ibo/NaCl *vs* unlesioned p= 0.0097 ##  Ibo/remi *vs* Ibo/NaCl p= 0.0078 ** |
|  | Negative geotaxis of female mice with ibotenate  (Fig. 5B) | Two-way ANOVA, Bonferroni post-test | Unlesionned n=17  Ibo/ NaCl n=17  Ibo/ remi n=17 | **Two-way ANOVA [F(DFn, DFd)]**  Interaction F(4, 144)= 1.183 p= 0.3208  Treatment factor F(2, 144)= 16.44 p< 0.0001  Postnatal age factor F(2, 144)= 6.085 p= 0.0029  **P6**  Ibo/NaCl *vs* unlesioned  p= 0.0002 ###  Ibo/remi *vs* Ibo/NaCl p= 0.0021**  **P7**  Ibo/NaCl *vs* unlesioned  p= 0.0005 ### |
|  | Latency before falling of female mice with ibotenate  (Fig. 5C) | Two-way ANOVA, | Unlesionned n=20  Ibo/ NaCl n=20  Ibo/ remi n=20 | **Two-way ANOVA [F(DFn, DFd)]**  Interaction F(4, 173)= 0.1337 p= 0.9698  Treatment factor F(2, 173)= 5.384 p= 0.0054  Postnatal age factor F(2, 173)= 9.735 p< 0.0001 |
|  | Latency before falling of female mice with ibotenate  (Fig. 5D) | Two-way ANOVA | Unlesionned n=17  Ibo/ NaCl n=17  Ibo/ remi n=17 | **Two-way ANOVA [F(DFn, DFd)]**  Interaction F(4, 145)= 0.6618 p= 0.6195  Treatment factor F(2, 145)= 0.6646 p= 0.5160  Postnatal age factor F(2, 145)= 7.392 p= 0.0009 |
| 12  (7 litters) | Negative geotaxis of unlesioned male mice  (Fig. 6A) | Two-way ANOVA | untreated n=11  NaCl n= 13  remi n= 13 | **Two-way ANOVA [F(DFn, DFd)]**  Interaction F(4, 102)= 1.305 p= 0.2731  Treatment factor F(2, 102)= 0.9275 p= 0.3988  Postnatal age factor F(2, 102)= 1.219 p= 0.2998 |
|  | Negative geotaxis of unlesioned female mice (Fig. 6B) | Two-way ANOVA | untreated n=15  NaCl n= 15  remi n= 16 | **Two-way ANOVA [F(DFn, DFd)]**  Interaction F(4, 132)= 0.6586 p= 0.6218  Treatment factor F(2, 132)= 1.234 p= 0.2944  Postnatal age factor F(2, 132)= 0.3542 p= 0.7024 |
|  | Latency before falling of unlesioned male mice (Fig. 6C) | Two-way ANOVA | untreated n=11  NaCl n= 13  remi n= 13 | **Two-way ANOVA [F(DFn, DFd)]**  Interaction F(4, 102)= 0.5955 p= 0.6667  Treatment factor F(2, 102)= 0.03219 p= 0.9683  Postnatal age factor F(2, 102)= 1.080 p= 0.3433 |
|  | Latency before falling of unlesioned female mice (Fig. 6D) | Two-way ANOVA | untreated n=15  NaCl n= 15  remi n= 16 | **Two-way ANOVA [F(DFn, DFd)]**  Interaction F(4, 128)= 1.149 p= 0.3365  Treatment factor F(2, 128)= 0.04003 p= 0.9608  Postnatal age factor F(2, 128)= 5.034 p= 0.0079 |
| 13  (7 litters) | Spontaneous motor activity at P18 in males with ibotenate  (Fig. 7A-B) | Two-way ANOVA | Ibo/ NaCl n=20  Ibo/ remi n=20 | **Fig. 7A Distance covered**  **Two-way ANOVA** **[F(DFn, DFd)]**  Interaction F(2, 114)= 0.2331 p= 0.7925  Treatment factor F(1, 114)= 0.08334 p= 0.7733  Time periods factor F(2, 114)= 4.001 p= 0.0209  **Fig. 7B Time spent in the center**  **Two-way ANOVA [F(DFn, DFd)]**  Interaction F(4, 190)= 1.492 p= 0.2061  Treatment factor F(1, 190)= 1.252 p= 0.2646  Time periods factor F(4, 190)= 0.6640 p= 0.6178 |
|  | Spontaneous motor activity at P18 in females with ibotenate  (Fig. 7C-D) | Two-way ANOVA | Ibo/ NaCl n=18  Ibo/ remi n=18 | **Fig. 7C Distance covered**  **Two-way ANOVA [F(DFn, DFd)]**  Interaction F(2, 102)= 0.07963 p= 0.9235  Treatment factor F(1, 102)= 2.744 p= 0.1007  Time periods factor F(2, 102)= 5.191 p= 0.0071  **Fig. 7D Time spent in the center**  **Two-way ANOVA [F(DFn, DFd)]**  Interaction F(4, 170)= 0.8669 p= 0.4851  Treatment factor F(1, 170)= 7.958 p= 0.0054  Time periods factor F(4, 170)= 0.3154 p= 0.8674 |
| 14  (7 litters) | Weight intake in males with ibotenate  (Supplementary Fig. 1A) | Two-way ANOVA | Ibo/ NaCl n=21  Ibo/ remi n=21 | **Two-way ANOVA [F(DFn, DFd)]**  Interaction F(10, 446)= 0.3494 p= 0.9697  Treatment factor F(1, 446)= 17.89 p< 0.0001  Postnatal age factor F(10, 446)= 231.7 p< 0.0001 |
|  | Weight intake in females with ibotenate  (Supplementary Fig. 1B) | Two-way ANOVA | Ibo/ NaCl n=17  Ibo/ remi n=17 | **Two-way ANOVA [F(DFn, DFd)]**  Interaction F(10, 351)= 0.1195 p= 0.9996  Treatment factor F(1, 351)= 2.172 p= 0.1414  Postnatal age factor F(10, 351)= 143.0 p< 0.0001 |
| 15  (7 litters) | Weight intake in unlesioned males  (Supplementary Fig. 1C) | Two-way ANOVA | NaCl n=13  remi n=13 | **Two-way ANOVA [F(DFn, DFd)]**  Interaction F(10, 264)= 0.07752 p= 0.9999  Treatment factor F(1, 264) = 0.006961 p= 0.9336  Postnatal age factor F(10, 264)= 181.4 p< 0.0001 |
|  | Weight intake in unlesioned females  (Supplementary Fig. 1D) | Two-way ANOVA | NaCl n=15  remi n=15 | **Two-way ANOVA [F(DFn, DFd)]**  Interaction F(10, 308)= 0.09283 p= 0.9999  Treatment factor F(1, 308)= 3.312 p= 0.0698  Postnatal age factor F(10, 308)= 218.4 p< 0.0001 |
| 16  (7 litters) | Spontaneous motor activity at P18 in unlesioned males  (Supplementary Fig. 2 A-B) | Two-way ANOVA | NaCl n=10  remi n=12 | **Sup. Fig. 2A Distance covered**  **Two-way ANOVA [F(DFn, DFd)]**  Interaction F(2, 60)= 0.7720 p= 0.4666  Treatment factor F(1, 60)= 0.03249 p= 0.8576  Time periods factor F(2, 60)= 6.852 p= 0.0021  **Sup. Fig. 2B Time spent in the center**  **Two-way ANOVA [F(DFn, DFd)]**  Interaction F(4, 105)= 1.099 p= 0.3611  Treatment factor F(1, 105)= 2.196 p= 0.1413  Time periods factor F(4, 105)= 0.2626 p= 0.9014 |
|  | Spontaneous motor activity at P18 in unlesioned females (Supplementary Fig. 2 C-D) | Two-way ANOVA | NaCl n=15  remi n=13 | **Sup. Fig. 2C Distance covered**  **Two-way ANOVA [F(DFn, DFd)]**  Interaction F(2, 78)= 0.2705 p= 0.7637  Treatment factor F(1, 78)= 0.7674 p= 0.3837  Time periods factor F(2, 78)= 10.81 p< 0.0001  **Sup. Fig. 2D Time spent in the center**  **Two-way ANOVA [F(DFn, DFd)]**  Interaction F(4, 130)= 0.2370 p= 0.9170  Treatment factor F(1, 130)= 0.1506 p= 0.6986  Time periods factor F(4, 130)= 0.4126 p= 0.7993 |
