# Supplementary material for: Beneficial Effects of Remifentanil Against Excitotoxic Brain Damage in Newborn Mice
Source: Front Neurol. 2019 Apr 24;10:407. doi: 10.3389/fneur.2019.00407 (PMC6491788; doi:10.3389/fneur.2019.00407)
Supplement: Supplementary Table 2 — Survival rates of NaCl- and remifentanil-treated pups injected at P2 with ibotenate at 5 days post-lesion. [file Table_2.DOCX]

Supplementary Table 2: Survival rates of NaCl- and remifentanil-treated pups injected at P2 with ibotenate at 5 days post-lesion.

|  | **5-day survival rate (%)** | | |
| --- | --- | --- | --- |
|  | **Ibo/NaCl** | **Ibo/remi** | ***p*** |
| **Males + Females** | 86 (57/66) | 91 (62/68) | 0,422 |
| **Males** | 83 (30/36) | 91 (34/37) | 0,3081 |
| **Females** | 90 (27/30) | 90 (28/31) | 1 |

**^§^** n/N: n represents number of survivors; N the number of injected pups (Fisher’s exact test)
